# Evidence-based decision-making in infectious diseases epidemiology, prevention and control: matching research questions to study designs and quality appraisal tools

**DOI:** 10.1186/1471-2288-14-69

**Published:** 2014-05-21

**Authors:** Thomas Harder, Anja Takla, Eva Rehfuess, Alex Sánchez-Vivar, Dorothea Matysiak-Klose, Tim Eckmanns, Gérard Krause, Helena de Carvalho Gomes, Andreas Jansen, Simon Ellis, Frode Forland, Roberta James, Joerg J Meerpohl, Antony Morgan, Holger Schünemann, Teun Zuiderent-Jerak, Ole Wichmann

**Affiliations:** 1 Robert Koch Institute, Berlin, Germany; 2 Institute of Medical Informatics, Biometry and Epidemiology, University of Munich, Munich, Germany; 3 Health Protection Scotland (HPS) and the Scottish Health Protection Network (HPN), Glasgow, UK; 4 Helmholtz Centre for Infection Research, Braunschweig, Germany; 5 European Centre for Disease Prevention and Control (ECDC), Stockholm, Sweden; 6 National Institute for Health and Care Excellence (NICE), London, UK; 7 Royal Tropical Institute, Amsterdam, The Netherlands; 8 Norwegian Institute of Public Health, Oslo, Norway; 9 Scottish Intercollegiate Guidelines Network (SIGN), Edinburgh, UK; 10 German Cochrane Center, University Medical Center Freiburg, Freiburg, Germany; 11 Departments of Clinical Epidemiology, Biostatistics & Medicine, McMaster University Health Sciences Centre, Hamilton, ON, Canada; 12 Department of Technology and Social Change, Linköping University, Linköping, Sweden; 13 Immunization Unit, Department for Infectious Disease Epidemiology, Robert Koch Institute, Seestrasse 10, Berlin 13353, Germany

**Keywords:** Evidence-based public health, Quality appraisal tools, Risk of bias, Study designs, Infectious disease prevention and control

## Abstract

**Background:**

The Project on a Framework for Rating Evidence in Public Health (PRECEPT) was initiated and is being funded by the European Centre for Disease Prevention and Control (ECDC) to define a methodology for evaluating and grading evidence and strength of recommendations in the field of public health, with emphasis on infectious disease epidemiology, prevention and control. One of the first steps was to review existing quality appraisal tools (QATs) for individual research studies of various designs relevant to this area, using a question-based approach.

**Methods:**

Through team discussions and expert consultations, we identified 20 relevant types of public health questions, which were grouped into six domains, i.e. characteristics of the pathogen, burden of disease, diagnosis, risk factors, intervention, and implementation of intervention. Previously published systematic reviews were used and supplemented by expert consultation to identify suitable QATs. Finally, a matrix was constructed for matching questions to study designs suitable to address them and respective QATs. Key features of each of the included QATs were then analyzed, in particular in respect to its intended use, types of questions and answers, presence/absence of a quality score, and if a validation was performed.

**Results:**

In total we identified 21 QATs and 26 study designs, and matched them. Four QATs were suitable for experimental quantitative study designs, eleven for observational quantitative studies, two for qualitative studies, three for economic studies, one for diagnostic test accuracy studies, and one for animal studies. Included QATs consisted of six to 28 items. Six of the QATs had a summary quality score. Fourteen QATs had undergone at least one validation procedure.

**Conclusions:**

The results of this methodological study can be used as an inventory of potentially relevant questions, appropriate study designs and QATs for researchers and authorities engaged with evidence-based decision-making in infectious disease epidemiology, prevention and control.

## Background

### Evidence-based medicine and evidence-based public health

The fallacies of relying solely on expert opinion to establish best practice in clinical decision-making and public health policies are now well exposed globally [1,2]. It is now standard practice in guideline development to draw on systematic reviews. With regard to interventions, randomized controlled trials (RCTs) are the gold standard due to their capacity to minimize bias, if well conducted. Systematic reviews of RCTs are therefore commonly used in decision-making. However, many questions that play an important role in decision-making –especially those not directly concerning the effectiveness and safety of an intervention– have not been and partly cannot be addressed by RCTs, and evidence from well-conducted observational studies becomes important for decision-making. Particularly in public health quasi-experimental designs have been used to evaluate population-level effects, such as controlled before-and-after studies, stepped wedge designs, and interrupted time series. In areas for which well conducted experimental trials are missing and pressure exists to make decisions in due time, guidance developers and decision-makers need to rely on the best available evidence, and guidance is needed on how to select and critically appraise different types of evidence to support rigorous and transparent decision-making [3].

In infectious disease prevention and control, specialists also need to consider what consequences a public health intervention might have at population level [4-7], e.g. on spread of a pathogen in the total population. In the context of developing recommendations for infectious disease prevention and control in particular, several relevant questions cannot (easily) be addressed by RCTs. Challenges include assessing population-level effects (e.g. serotype-replacement following vaccine introduction, development of antibiotic resistance, or herd protection through reduced pathogen transmission) and long-term aspects of the intervention (e.g. the need for a booster vaccination ten years after primary vaccination), but also data on the disease burden in a given population, cost-of-illness, risk factors for infection or increased disease severity, or the mode of transmission of a newly recognized disease or during a nosocomial outbreak. Therefore, evidence informing these potentially relevant questions needs to take into account summaries from different sorts of research, including case-control studies, incidence studies, passively collected surveillance data, case series, outbreak investigations, and single case reports. In adopting the idea of “best available evidence” [6], tracing the full causal chain from intervention to outcomes within a given context requires a variety of fit-for-purpose methods from multiple disciplines.

### The PRECEPT approach

More recently developed evidence appraisal and grading systems are designed to incorporate information from studies with different designs, including RCTs as well as observational studies. The most prominent system developed by the Grading of Recommendations Assessment, Development and Evaluation Working Group (GRADE) [8,9] has already been widely applied not only in clinical medicine, but also in the context of public health interventions by many public health institutions [10-12], although several agencies explicitly opted against the GRADE approach [13]. A working group, which was established by the European Centre for Disease Prevention and Control (ECDC), discussed the application of GRADE in infectious disease prevention and control [14].

The Project on a Framework for Rating Evidence in Public Health (PRECEPT) was initiated by ECDC in 2012 to build upon the work done by this working group. It aims to define a framework for evaluating and grading evidence and strength of recommendations in the field of infectious disease epidemiology, prevention and control. An important challenge is that PRECEPT, in contrast to other frameworks in the field of evidence-based medicine, is not restricted to the appraisal of interventions. Rather, since relevant information in the field of application of PRECEPT comes from non-interventional studies, such as cross-sectional studies, surveillance systems and case series, it aims at assessing evidence from intervention as well as non-intervention studies.

Before the quality of a “body of evidence” consisting of multiple studies on a certain outcome can be assessed, it is necessary to evaluate the methodological quality of the single studies that constitute it. Quality appraisal tools (QATs) are designed to evaluate the quality of an individual study. As a first and necessary step, we therefore decided to review already existing QATs with respect to their usefulness across a range of commonly encountered/critical questions in the field of infectious disease prevention and control.

## Methods

### Objectives

**Figure 1:**
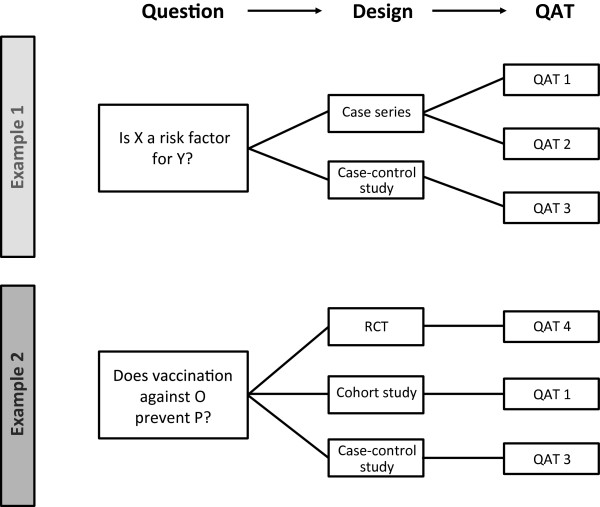
**Review of quality appraisal tools (QATs).** The approach starts with questions which are relevant to infectious disease epidemiology, prevention and control. Study designs are matched against these questions, followed by identification of QATs.

Given the above-mentioned challenges in appraising evidence from a great variety of interventional as well as non-interventional studies, we decided to use a novel approach to identify QATs which are suitable for the project. The approach uses relevant questions as starting point. Against these questions, study designs which are able to address the questions are mapped. Finally, QATs addressing the study designs were identified, using a systematic review as starting point. The approach is illustrated in Figure 1.

Accordingly, this methodological study had the following objectives:

1) To identify relevant questions which are commonly addressed during decision-making processes in the field of infectious disease prevention and control.

2) To map appropriate study designs to these questions.

3) To identify and characterize existing quality appraisal tools (QATs) which match the respective study designs and are useful in the context of infectious disease prevention and control.

### Scope and definitions

Prerequisite for the conduct of a review on QATs is to define the meaning of the term “quality”. According to the Agency for Healthcare Research and Quality (AHRQ), “methodological quality” can be defined as the extent to which a study’s design, conduct, and analysis has minimized selection, measurement, and confounding biases [15,16]. Thereby, this definition refers to internal validity of a study or risk of bias [16]. This perspective is also used by other public health agencies, such as the Canadian Agency for Drugs and Technologies in Health (CADTH), which defines methodological quality as “risk of bias” or “internal validity” [17]. However, it is often of equal importance to examine external validity, defined as the degree to which results of a study may be generalized to other groups or populations [18]. Therefore, some QATs commonly employed are also concerned with external validity. For the purposes of this study, we adopted an approach that considers both internal and external validity: We primarily focused on risk of bias but highlighted where QATs also addressed external validity.

It is crucial to separate methodological quality, that is, the quality of the design, conduct and analysis of a study, from transparency and completeness of reporting [19], since it has been shown that studies which have similar reporting quality may be different regarding methodological quality [20]. Therefore, consensus statements and related checklists, which aim to increase the quality of reporting but do not primarily assess the quality of the underlying study, such as CONSORT for randomized trials [21] or STROBE for observational studies [22], were not considered for this review. According to the definition used by the CADTH report [17], quality appraisal tools (QATs) are applied to transparently evaluate the quality of individual studies.

### Identification of relevant questions and study designs

The project team began with the development of a draft matrix, which comprised the potentially relevant questions grouped in domains. The initial starting point for the list of questions was the Standard Operating Procedure (SOP) of the German Standing Committee on Vaccinations [23]. This document was used as starting point since the SOP contains a comprehensive list of questions relevant for evidence appraisal and decision making in the field of infectious disease prevention, with a particular focus on vaccination, that has been developed by a multidisciplinary team of researchers based on the results of an international expert workshop [10]. This matrix was circulated among the authors for comments and suggestions, leading to the identification of additional questions. Four authors (TH, AT, ER and OW) developed a proposal for grouping of questions in domains. This proposal was again circulated among the authors for suggestions and comments.

In the second step of matrix building, each of the scientists involved was asked to map study designs against the identified questions. All types of study designs (quantitative and qualitative research) were considered. To define the study designs, we primarily used the list provided by the NICE Public Health methods manual [13]. In the third step, this list of questions and study designs was supplemented with appropriate QATs, which were identified as described below (Figure 1).

### Identification of QATs

As a starting point for our approach, a current (publication date: July 2012) systematic review on reviews on QATs [17] was used. The rationale behind this approach was to apply an efficient and time-saving strategy. The QATS identified in this report were considered for the matrix. Additionally, we used this systematic review [17] as the basis for snowballing techniques, i.e. pursuing references of references [24] to identify other potentially relevant systematic reviews of QATs. This process led to the identification of further seven systematic reviews [16,19,25-29]. The resulting body of a total of eight systematic reviews [16,17,19,25-29] of QATs was then screened for relevant QATs which addressed at least one of the identified study designs. These QATs were considered for the matrix.

QATs which met the inclusion criteria as given below were extracted from these reviews. However, since QATs identified by this approach covered only a minority of questions and designs, we asked members of the study team to name additional tools.

### Eligibility criteria for QATs

According to the above-mentioned definitions [16,17], a tool was defined to be a QAT if it is intended to appraise the methodological quality (internal validity) of a study.

Each identified QAT was evaluated by two independent reviewers (T.H. and A.T.) for its eligibility. QATs were included if they fulfilled the following a priori defined inclusion criteria:

1) The tool has been published (either in a journal or on a website).

2) The tool covers at least one study design of the matrix.

3) The tool is suitable for rating study quality, that is, a list of methodological items is given that have to be answered or assessed.

4) The tool was developed for general use (not for a specific study).

5) The tool has undergone at least one validation procedure (e.g., inter-rater reliability).

The following example might illustrate this approach. The Cochrane Collaboration’s tool for assessing risk of bias fulfilled all five inclusion criteria: 1) The tool has been published in the Cochrane Handbook and in a journal article. 2) The tool covers randomized controlled trials being one of the relevant study designs. 3) Study quality (or risk of bias) is rated by addressing six domains (selection bias, performance bias, detection bias, attrition bias, reporting bias, other bias) and making the judgement of “low risk of bias”, “high risk of bias” or “unclear risk of bias”. 4) The tool was developed to be applied to all randomized controlled trials. 5) The tool has been validated regarding interrater reliability (for details, see Appendix B).

The reviewers made exceptions from these eligibility criteria to arrive at a more comprehensive list of QATs, in particular when there was a lack of other QATs for a defined study design or when a QAT was very frequently used in public health. In such cases, a note was added to the description of the QAT (see below).

The following example might illustrate such an exception. The checklist developed by the Cochrane Effective Practice and Organization of Care (EPOC) group did only fullfil three out of five of the inclusion criteria: It has not been published so far (criterium 1) and has not been validated (criterium 5). However, the QAT was included in our matrix since it is very frequently used in public health.

### Data extraction for QATs

From each publication of a QAT, we extracted the following information:

1) For which study design(s) is the instrument intended to be used?

2) What was the primary purpose for which the instrument was developed?

3) How many questions does the instrument comprise?

4) Are questions grouped in domains?

5) What are the main contents of the questions (or, which domains are covered)?

6) What types of questions are used (e.g., predefined, open)?

7) Does the instrument include a quality summary score?

8) If 7) is answered with yes: how is the score calculated?

9) According to the authors, how much time is needed to apply the tool on average?

10) How was the instrument validated?

11) What was the main result of the validation procedure?

Results of this data extraction were summarized in an abstract for each QAT.

### Analysis

The extracted information on relevant questions, study designs and QATs was analyzed as follows:

• A matrix was constructed that mapped study designs to relevant questions, and QATs to study designs.

• The data extracted from the original descriptions of the QATs was summarized in one abstract per QAT.

• The number of questions/items of each QAT, information on the structure (checklist, scale, summary score) and validation of each QAT was summarized across all QATs.

## Results

### Relevant questions

We identified a total of 20 questions of potential relevance during decision-making processes in the field of infectious disease epidemiology, prevention and control. These questions were grouped into 6 domains (A – F):

Domain A: characteristics of the pathogen

What are the …

1) Characteristics of the pathogen (pathogenicity, virulence, reservoir)?

2) Subtypes, serotypes and local epidemiology (incl. seasonality) of the pathogen?

3) Modes of transmission?

Domain B: burden of disease

What is/are the …

4) Incidence of the disease?

5) Prevalence/seroprevalence of the disease/pathogen?

6) Consequences or sequelae of the disease:

6.1 Hospitalization rate?

6.2 Mortality?

6.3 Complication rate (acute)?

6.4 Rate of disabilities (chronic)?

7) Perception of the disease in the target population?

Domain C: diagnosis

What is the …

8) Sensitivity of tests?

9) Specificity of tests?

Domain D: risk factors

10) **What are risk factors for …**

10.1 Transmission?

10.2 Colonisation?

10.3 Infection/disease?

10.4 Exacerbation/complications?

Domain E: intervention

11) **What are effects of the intervention at the individual/population level in terms of …**

11.1 Efficacy (under controlled conditions)?

11.2 Direct/indirect/overall effectiveness (under uncontrolled conditions)?

11.3 Surrogate markers for 11.1 and 11.2?

11.4 Risk of adverse events/harms?

Domain F: implementation of intervention (or diagnostic measure) in the population

Is the intervention (or diagnostic measure) …

12) Feasible to implement?

13) Cost-effective?

14) Acceptable to most relevant stakeholders?

15) Equitable or equity-enhancing?

What are …

16) Enablers and barriers to success?

17) Coverage rates needed to induce positive population level effects?

How …

18) Shall the advice be communicated (incl. the hard to reach)?

19) Shall population preferences be weighted and valued?

20) Effective and implementable are alternative measures?

### Study designs

The following 26 different study designs (grouped into six categories) were considered to be potentially able to inform the relevant questions:

I) **Quantitative studies - experimental:**

a) Randomized controlled trial (RCT)

b) Cluster-randomized controlled trial (cRCT)

c) Non-randomized controlled trial (NRCT) and other quasi-experimental study

d) Controlled before-and-after study

II) **Quantitative studies - observational:**

e) Uncontrolled before-and-after study

f) Interrupted time series (ITS)

g) Cohort study

h) Case-control study

i) Ecological study (correlation study)

j) Cross-sectional study/Surveillance

k) Self-controlled case series

l) Case series

m) Case report (single case study)

III) **Qualitative studies:**

n) Document analysis

o) Focus groups

p) Interview study

q) Observation and participant observation

r) Process evaluation

IV) **Economic studies/Mathematical modeling studies:**

s) Cost-benefit analysis

t) Cost-consequence analysis

u) Cost-effectiveness analysis

v) Cost-utility analysis

w) Non-economic modeling study

V) **Diagnostic test accuracy studies**

VI) **Non-epidemiological evidence**

x) Animal study

y) Other laboratory study

For definitions of the study designs, see Appendix A (Glossary of study designs).

### Quality appraisal tools (QATs)

**Figure 2:**
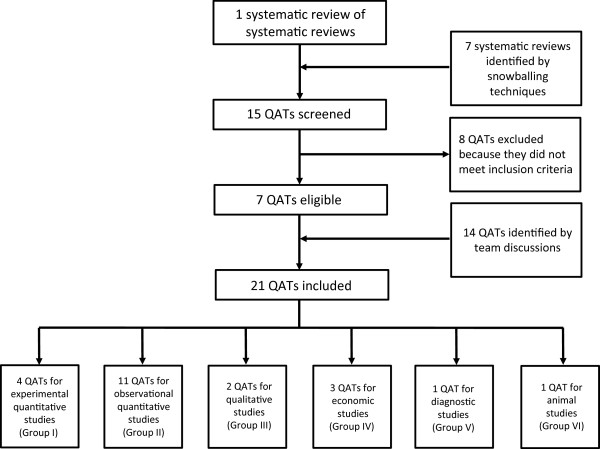
Flow chart: identification and selection of quality appraisal tools (QATs) during the review process.

The review process led to the identification of a total of 21 QATs [30-50]. The selection process is illustrated as a flow chart in Figure 2. A total of seven QATs could be extracted from the above-mentioned reviews and met criteria for eligibility. These seven QATs, however, covered only a minority of questions and study designs addressed in the matrix. Discussions in the project team identified an additional 14 relevant QATs, leading to a total of 21 QATs. According to the six categories of relevant study designs described above, four of these QATs are applicable to experimental quantitative study designs, eleven are intended for observational quantitative studies, two for qualitative studies, three for economic studies, one for diagnostic test accuracy studies and one for animal studies (Figure 2).

In Table 1, these QATs were mapped against the respective questions and study designs. To the majority of the 20 questions, more than one study design could be matched. For example, question 11 (What are the effects of intervention?) can be addressed by 10 different study designs, ranging from experimental studies (RCT, cluster-randomized trial) to observational studies (case-control study, cohort study etc.). For some questions, however, only one study design was judged to be suitable: Question 5 (What is the prevalence of the disease?) can only be addressed by cross-sectional studies. Similar observation was made when QATs were matched to study designs. For the majority of study designs, we found more than one QAT to be applicable. For example, cohort studies and case-control studies can be assessed by five different QATs each. However, for some designs, such as animal studies and cost-utility analyses, we identified only one eligible QAT per study design.

**Table 1:** Tabulation of questions, respective study designs and quality appraisal tools which are relevant in the field of infectious disease epidemiology, prevention and control

**Domain**^**1**^	**No.**	**Question**	**Study design**	**Quality appraisal tools (Reference)**^**2**^
A	1	Characteristics of the pathogen?	Laboratory study	Van der Worp [37]^3^
A	2	Subtypes, serotypes and local epidemiology of the pathogen?	Laboratory study	Van der Worp [37]
			Cross-sectional study	Al-Jader [33], Loney [32], Hoy [45], Cho [30], NICE [47]
A	3	Modes of transmission?	Animal study	Van der Worp [37]
			Cohort study	Downs [31], SIGN [40], Cho [30], EPHPP [38], NOS [44]
			Case series	Cho [30]
			Case-control study	Downs [31], SIGN [41], Cho [30], EPHPP [38], NOS [44]
B	4	Incidence of the disease?	Cohort study	Downs [31], SIGN [40], Cho [30], EPHPP [38], NOS [44]
B	5	Prevalence/seroprevalence of the disease?	Cross-sectional study	Al-Jader [33], Loney [32], Hoy [45], Cho [30], NICE [47]
B	6	Consequences or sequelae of the disease (hospitalization/mortality/complications/disabilities)?	Cohort study	Downs [31], SIGN [40], Cho [30], EPHPP [38], NOS [44]
			Case series	Cho [30]
			Case report	Cho [30]
B	7	Perception of the disease in the target population?	Cross-sectional study	Al-Jader [33], Loney [32], Hoy [45], Cho [30], NICE [47]
			Cohort study	Downs [31], SIGN [40], Cho [30], EPHPP [38], NOS [44]
			Focus groups	NICE [48], CASP [36]
			Interview study	
C	8	Sensitivity of tests?	Diagnostic test accuracy study	SIGN [42]
C	9	Specificity of tests?	Diagnostic test accuracy study	SIGN [42]
D	10	Risk factors (for transmission/colonization/infection/disease/exacerbation/complication)?	Cohort study	Downs [31], SIGN [40], Cho [30], EPHPP [38], NOS [44]
			Case-control study	Downs [31], SIGN [41], Cho [30], EPHPP [38], NOS [44]
			Ecological study	NICE [47]
			Cross-sectional study	Al-Jader [33], Loney [32], Hoy [45], Cho [30], NICE [47]
			Animal study	Van der Worp [37]
E	11	Effects of intervention (in terms of efficacy/effectiveness/surrogate markers/adverse events/harms)?	Controlled before-and-after study	Downs [31], NICE [46], EPHPP [38]
			RCT	Cochrane [35], Downs [31], SIGN [39], NICE [46], EPHPP [38]
			NRCT	Downs [31], NICE [46], EPHPP [38]
			Cluster-randomized trial	Downs [31], NICE [46], EPHPP [38]
			Cohort study	Downs [31], SIGN [40], Cho [30], EPHPP [38], NOS [44]
			Case-control study	Downs [31], SIGN [41], Cho [30], EPHPP [38], NOS [44]
			Uncontrolled before-and-after study	Downs [31], NICE [46], EPHPP [38]
			Ecological study	NICE [47]
			Interrupted time series	EPOC [50], EPHPP [38]
			Self-controlled case series	Cho [30], NOS [44], SIGN [41]
F	12	Feasible to implement?	Cross-sectional study	Al-Jader [33], Loney [32], Hoy [45], Cho [30], NICE [47]
			Cohort study	Downs [31], SIGN [40], Cho [30], EPHPP [38], NOS [44]
			Focus groups	NICE [48], CASP [36]
			Interview study	
F	13	Cost-effectiveness of the intervention?	Cost-effectiveness (-benefit, -consequence) analysis	SIGN [43], NICE [49], QHES [34]
			Cost-utility analysis	QHES [34]
F	14	Acceptable to stakeholders?	Focus groups	NICE [48], CASP [36]
			Interview study	
			Cross-sectional study	Al-Jader [33], Loney [32], Hoy [45], Cho [30], NICE [47]
F	15	Equitable?	Cross-sectional study	Al-Jader [33], Loney [32], Hoy [45], Cho [30], NICE [47]
			Cohort study	Downs [31], SIGN [40], Cho [30], EPHPP [38], NOS [44]
F	16	Enablers/barriers to success?	Cross-sectional study	Al-Jader [33], Loney [32], Hoy [45], Cho [30], NICE [47]
			Interview study (observation)	NICE [48], CASP [36]
			Document analysis	
			Focus groups	
			Process evaluation	
F	17	Coverage rates for positive population level effects?	Cohort study	Downs [31], SIGN [40], Cho [30], EPHPP [38], NOS [44]
			Non-economic modeling study	SIGN [43], NICE [49]
F	18	Communication of advice?	RCT	Cochrane [35], Downs [31], SIGN [39], NICE [46], EPHPP [38]
			NRCT	Downs [31], NICE [46], EPHPP [38]
			Cohort study	Downs [31], SIGN [40], Cho [30], EPHPP [38], NOS [44]
			Cross-sectional study	Al-Jader [33], Loney [32], Hoy [45], Cho [30], NICE [47]
			Focus groups	NICE [48], CASP [36]
			Interview study	
F	19	Weighing and valuing of population preferences?	Cross-sectional study	Al-Jader [33], Loney [32], Hoy [5], Cho [30], NICE [47]
			Focus groups	NICE [48], CASP [36]
			Interview study	
F	20	Effectiveness of alternative measures?	Controlled before-after study	Downs [31], NICE [46], EPHPP [38]
			RCT	Cochrane [35], Downs [31], SIGN [39], NICE [46], EPHPP [38]
			NRCT	Downs [31], NICE [46], EPHPP [38]
			Cluster-randomized trial	Downs [31], NICE [46], EPHPP [38]
			Cohort study	Downs [31], SIGN [40], Cho [30], EPHPP [38], NOS [44]
			Case-control study	Downs [31], SIGN [41], Cho [30], EPHPP [38], NOS [44]
			Uncontrolled before-after study	Downs [31], NICE [46], EPHPP [38]
			Ecological study	NICE [47]
			Interrupted time series	EPOC [50], EPHPP [38]
			Self-controlled case series	Cho [30], NOS [44], SIGN [41]

^1^Domains A-F refer to the following domains of questions described under Results: A – Characteristics of the pathogen; B – Burden of disease; C – Diagnosis; D – Risk factors; E – Intervention; F – Implementation of intervention.

^2^See footnote 2 of Table 2 for complete names of the included QATs.

^3^For alternative QATs for animal studies, see the review by Krauth et al. [51] published after completion of our literature search.

The following two examples should illustrate the approach of the matrix:

• For the research question “Is neonatal sepsis a risk factor for neurodevelopmental delay?”, the user would choose domain D, question no. 10 (risk factors). Five different study designs are suggested to be applicable: cohort study, case-control study, ecological study, cross-sectional study and animal study. If the user has identified a cohort study, he/she is guided by the matrix to use one out of five different QATs to assess the methodological quality of the study (Downs, SIGN, Cho, EHPP or NOS).

• For the research question “What is the prevalence of neonatal sepsis?”, the user would choose domain B, question 5 (prevalence). He/she is guided to cross-sectional studies as the appropriate study design. For methodological quality appraisal of this type of studies, five QATs are suggested (Al-Jader, Loney, Hoy, Cho and NICE).

Table 2 shows a cross-tabulation of all QATs (rows) against all study designs (columns) which were considered. The table is intented to guide users who are looking for an appropriate QAT for a particular study with a given design. Study designs are ordered alphabetically in columns from the left-hand to the right-hand side. QATs are shown in the order of appearance in Table 1 from top to bottom. Nine QATs are only applicable to a single study design, whereas 12 QATs can be used for more than one study design.

**Table 2:** Cross-tabulation of quality appraisal tools (QATs) against study designs

**QAT (Reference)**^**1**^	**Animal study**	**Before-and-after study (controlled)**	**Before-and-after-study (uncontrolled)**	**Case-control study**	**Case report**	**Case series**	**(Cluster) rRCT**	**Cohort study**	**Cost-effectiveness (-benefit, -consequence) analysis**	**Cost-utility analysis**	**Cross-sectional study**	**Diagnostic test accuracy study**	**Document analysis**	**Ecological study**	**Focus groups**	**(Individually) RCT**	**Interrupted time series**	**Interview study (Observation study)**	**Laboratory study**	**Non-economic modeling study**	**nRCT**	**Process evaluation**	**Self-controlled case series**
Van der Worp [37]	**X**																		**X**				
NICE (qualitative) [48]													**X**		**X**			**X**				**X**	
CASP [36]													**X**		**X**			**X**				**X**	
SIGN (diagnostic) [42]												**X**											
Cho [30]		**X**	**X**	**X**	**X**	**X**	**X**	**X**			**X**					**X**					**X**		**X**
Hoy [45]											**X**												
Al-Jader [33]											**X**												
SIGN (cohort) [40]								**X**															
NOS [44]				**X**				**X**															**X**
EPOC [50]																	**X**						
SIGN (case-control) [41]				**X**																			**X**
NICE (intervention) [46]							**X**									**X**					**X**		
Cochrane [35]																**X**							
SIGN (RCT) [39]																**X**							
NICE (correlation) [47]														**X**									
Downs et al. [31]		**X**	**X**	**X**			**X**	**X**								**X**					**X**		
Loney et al. [32]											**X**												
QHES [34]									**X**	**X**										**X**			
EPHPP [38]		**X**	**X**	**X**			**X**	**X**								**X**	**X**				**X**		
SIGN (economic) [43]									**X**											**X**			
NICE (economic) [49]									**X**											**X**			

^1^Complete names of the included QATs (in the order of appearance): Van der Worp: Aspects of study quality to be reported; NICE (qualitative): Quality appraisal checklist: qualitative studies; CASP: Critical appraisal skills programme tools; SIGN (diagnostic): Scottish Intercollegiate Guidelines Network (SIGN) checklist 5 (diagnostic studies); Cho: Methodologic quality instrument; Hoy: Tool to assess risk of bias in prevalence studies; Al-Jader: Quality scoring system for epidemiological surveys of genetic disorders; SIGN (diagnostic): Scottish Intercollegiate Guidelines Network (SIGN) checklist 3 (cohort studies); NOS: Newcastle-Ottawa Scale; EPOC: Cochrane Effective Practice and Organization of Care (EPOC) Groups risk of bias tool for interrupted time series; SIGN (case-control): Scottish Intercollegiate Guidelines Network (SIGN) checklist 4 (case-control studies); NICE (intervention): National Institute for Health and Care Excellence quality appraisal checklist for quantitative intervention studies; Cochrane: Cochrane risk of bias tool for RCTs; SIGN (RCT): Scottish Intercollegiate Guidelines Network (SIGN) checklist 2 (RCTs); NICE (correlation): National Institute for Health and Care Excellence quality appraisal checklist for quantitative studies reporting correlations and associations; Downs: Checklist for the assessment of the methodological quality of randomized and non-randomized studies of health care interventions; Loney: Guidelines for critically appraising studies of prevalence or incidence of a health problem; QHES: Quality of Health economic studies instrument; EPHPP: Effective Public Health practice projects quality assessment tool for quantitative studies; SIGN (economic): Scottish Intercollegiate Guidelines Network (SIGN) checklist 6 (economic studies); NICE (economic): National Institute for Health and Care Excellence quality appraisal checklist for economic evaluations.

In Table 3, basic information is provided regarding the content and validation of QATs. QATs are shown in the order of their appearance in Table 1. For each QAT, the number of questions/items is summarized. Furthermore, information is given whether the QAT is a checklist or a scale, whether it has a summary score and whether it has been validated so far. QATs had six to 28 items, with the majority having more than ten items. Five QATs were scales, while the remaining 16 were checklists. Six of the QATs had a summary quality score. Fourteen QATs had undergone at least one validation procedure. The approach, content and validation of each QAT are described in detail in the related Appendix B.

**Table 3:** Characteristics of included quality appraisal tools (QATs)

**QAT (Reference)**	**No. of questions or items**	**Checklist (C) or scale (S)?**	**Summary score? (yes/no)**	**Validation? (yes/no)**
Van der Worp [37]	9	C	No	No
NICE [47]	20	C	No	No
SIGN [39]	10	C	No	yes
Cho [30]	24	C	yes	Yes
Hoy [45]	10	C	No	Yes
Al-Jader [33]	9	S	Yes	Yes
SIGN [40]	16	C	No	Yes
NOS [44]	8	S	No	Yes
EPOC [50]	7	C	No	No
SIGN [41]	13	C	No	Yes
NICE [46]	27	C	No	No
Cochrane [35]	6	C	No	Yes
SIGN [42]	28	C	No	Yes
Loney [32]	8	S	Yes	Yes
Downs [31]	27	S	Yes	Yes
EPHPP [38]	20	C	Yes	Yes
CASP [36]	10	C	No	No
NICE [48]	15	C	No	No
SIGN [43]	20	C	No	Yes
NICE [49]	19	C	No	No
QHES [34]	16	S	Yes	Yes

## Discussion

So far, reviews of QATs have been conducted by using the study types they cover as a basis. For our methodological study we chose a new strategy and applied for the first time a question-based approach. Our study is intended to represent a starting point for comprehensive evidence-based decision making in infectious disease prevention and control through the formulation of a broad set of questions and matching these questions to the most appropriate evidence in terms of study design followed by assessing study quality.

One has to keep in mind, however, that there is no direct relation between a research question and a QAT. Rather, QATs are constructed to assess the methodological quality of a study which has a given study design. According to the key features of different study designs, different sources of bias arise. For example, the effectiveness of an intervention can be assessed by randomized controlled studies or by case-control studies. In the randomized controlled studies, inadequate blinding of participants is an issues that can be assessed by an appropriate QAT (e.g., the Cochrane risk of bias tool). However, if the same intervention question is addressed by a case-control study, blinding of participants does not play a role. Rather, other specific issues of case-control studies, such as adequate control selection, become important and are captured by appropriate QATs (e.g., the Newcastle-Ottawa Scale).

The strengths of our approach include the development of a comprehensive picture which covers the full process from question development to quality appraisal. Through multiple rounds of discussions and consultations, we identified a total of 20 questions considered most important for evaluating and grading evidence and strength of recommendations in the field of infectious disease epidemiology, prevention and control. The majority of these can be addressed by experimental and observational study designs.

For some questions the appraisal of non-epidemiological evidence was needed. We therefore also considered non-epidemiological study designs, such as qualitative studies. This type of studies should be considered if epidemiological studies are unlikely to provide useful information for a given research question. For example, as shown in Table 1 under Domain F, question 19, for weighing and valuing of population preferences, the conduct of focus groups might be appropriate. This will be particularly important in a situation when no prior information is available regarding such preferences and the researcher has to generate new hypotheses about them. Other relevant questions which can be answered by qualitative research include those about enablers and barriers to the success of an intervention (Domain F, question 16).

The first step in the identification of appropriate appraisal instruments was to consider review papers of QATs. The rationale behind this approach was to apply an efficient and time-saving strategy. We hereby decided to start with a very recent systematic review and to complement its findings with those from other systematic reviews, using snowballing techniques. Many of the published instruments focus on experimental study designs, but do not cover other relevant study designs, such as diagnostic studies or qualitative studies. Therefore, we had to use a second approach (expert consultations) to fill the gaps in our question matrix. In particular, the latter approach helped to identify QATs for study designs such as (controlled) before-and-after-studies and interrupted time series. These designs have in common that they are widely used in public health research, but are not as intensively reflected in the tradition of evidence-based medicine. The QATs identified and included in the matrix showed large variability regarding their length and complexity. Only two-thirds of the QATs were validated by the authors or other investigators. Moreover, validation procedures differed markedly, making comparisons regarding the validity of the QATs difficult.

During recent years, it has been intensively discussed whether or not QATs should have a summary score [52]. For this review, we considered QATs irrespective of whether they had such a score. However, for a comparison of evidence assessments based on different QATs for different study designs, a summary score might be useful. Whether or not the use of a score in a QAT is necessary for further development of PRECEPT will be one aspect to be discussed and evaluated later.

Limitations of our approach mainly regard the identification of appropriate QATs. Whereas for some very common study designs QATs could easily be identified based on systematic reviews, this was not feasible for other study designs and had to be done by team discussions. Furthermore, combining different QATs across different study designs may lead to problems in practice. It should therefore be evaluated whether “packages” of QATs developed within one framework (e.g. NICE (15)) may be preferable. Finally, one has to keep in mind that the choice of QAT may exert a significant influence on the result of the respective systematic review. This has been shown recently in a study demonstrating that results of sensitivity analyses according to study quality show considerable variability depending on which QAT was used to assess study quality in a meta-anaysis [53].

## Conclusions

Our question-centered review is the first that assembles QATs for all study designs considered relevant in the field of infectious diseases. Thereby, it adds to what is already known on QATs by providing a systematic overview on this topic, including a description and assessment of the included QATs. Our primary goal was to inform and establish the basis for the development of the PRECEPT evidence assessment framework. In addition, we provide an inventory of questions, study designs and quality appraisal tools to support public health researchers and authorities in assessing evidence when developing recommendations for infectious disease prevention and control. The inventory can easily be supplemented if new tools are published in the future. Next steps will be to integrate these findings into the PRECEPT framework, and to define a methodology how to assess bodies of evidence within the framework.

### Ethical approval

For this methodological review study no ethical approval was needed.

## Appendix A

### Glossary of study designs

Definitions were derived from the NICE manual [13] and additional references [18,54-57].

• *Animal study/other laboratory study*

A study in a population of laboratory animals that often uses conditions of animals analogous to conditions in humans to model processes that occur in human populations (analogous for other laboratory studies using cell culture etc).

• *Before-and-after study (controlled: experimental; uncontrolled: observational)*

An approach where the dependent variables are measured before and after an intervention has been delivered. The intervention can either be delivered by the investigator or by others (experimental vs observational before-and-after study).

• *Case-control study*

A comparative observational study in which the investigator selects people who have an outcome of interest (for example, developed a disease) and others who have not (controls), and then collects data to determine previous exposure to possible causes.

• *Case report*

Detailed description of a single patient or clinical case.

• *Case series*

A collection of patients with common characteristics used to describe aspects of a disease, diagnosis, treatment and prognosis.

• *Cohort study*

An observational study in which a group of people is observed over time in order to see who develops the outcome of interest.

• *Cost-benefit analysis*

An analysis which investigates whether all benefits outweigh all costs of an intervention.

• *Cost-consequence analysis*

An analysis which contrasts the resources and costs to the results of an activity, usually in table form.

• *Cost-effectiveness analysis*

An analysis which asks how one can maximize health (measured as clinical outcome) for available resources.

• *Cost-utility analysis*

A form of economic evaluation in which the outcomes of alternative procedures or programs are expressed in terms of a single “utility-based” unit of measurement.

• *Cross-sectional study*

An observational study in which the source population is examined to see what proportion has the outcome of interest, or has been exposed to a risk factor of interest, or both, at a fixed time point.

• *Diagnostic test accuracy study*

A study which determines the sensitivity and/or specificity of a diagnostic test or measure.

• *Document analysis*

A quantitative or qualitative approach that consists of the systematic reading and compiling of a body of texts, images, and symbolic matter.

• *Ecological study*

An observational study in which the units of analysis are populations or groups of people rather than individuals.

• *Focus groups*

A sample of people (usually a relatively homogeneous group drawn by purposive sampling) brought together to discuss a topic or issue with the aim of ascertaining the range and intensity of their views rather than arriving at a consensus.

• *Interrupted time series*

An approach in which multiple (more than two) observations are made on the same individuals, or groups of individuals, over time. Some authors demand that a defined number of data points have to be assessed before and after the intervention of interest (e.g., three before and three after).

• *Interview study (qualitative)*

A qualitative method of data collection where participant’s views are elicited via verbal interviews, consisting of mostly open-ended questions.

• *Non-economic modeling study (mathematical model)*

A study which uses a representation of a system, process, or relationship in mathematical form in which equations are used to simulate the behavior of the system or process under study (use of Bayesian or frequentist methods possible).

• *Non-randomized controlled trial (NRCT)*

An experimental study in which participants are allocated to receive either intervention or control (or comparison intervention) but the allocation is not randomized.

• *Observation/participant observation*

A qualitative methodology where the researcher is (or pretends to be) a member of the group being studied.

• *Process evaluation*

The systematic collection of information on a program’s inputs, activities, and outputs, as well as the program’s context and other key characteristics.

• *Randomized controlled trial (RCT)/cluster-randomized controlled trial*

An experimental study in which participants (or clusters) are randomly allocated to receive either intervention or control.

• *Self-controlled case series*

A study design which solely uses cases that function at the same time as their own controls by estimating the relative incidence of an acute event in their risk period (e.g. defined as during or after exposure) compared to the risk of an acute event in their control period.

## Appendix B

Description of included quality appraisal tools (QATs) (available upon request from the corresponding author).

## Competing interests

The authors declare that they have no competing interests.

## Authors’ contributions

TH, AT, ER, ASV and OW designed the study, performed the main analyses and drafted the discussion. AJ and HCG initiated the project and contributed to the study design and discussion. FF, JJM and HS contributed to background, data analyses and discussion. DMK, TE, GK, SE, RJ, AM and TZJ contributed to the discussion. All authors read and approved the final manuscript.

## Pre-publication history

The pre-publication history for this paper can be accessed here:

http://www.biomedcentral.com/1471-2288/14/69/prepub
